# Hydration of guanidinium depends on its local environment†
†Electronic supplementary information (ESI) available: Full citation for ref. 51, experimental details and reproducibility of the IRPD measurements, comparison between experimental IRPD spectra with harmonic IR spectra of energetic low lying isomers for [Gdm(H_2_O)_*n*_]^+^, *n* = 6–9, structures of the energetic low lying isomers of [Gdm(H_2_O)_*n*_]^+^, *n* = 6–9, and representative structures of [Gdm(H_2_O)_*n*_]^+^, [Na(H_2_O)_*n*_]^+^, [TMA(H_2_O)_*n*_]^+^, with *n* = 20 and 40. Quantitative comparison of the spectra for Gdm^+^, Na^+^ and TMA^+^ with *n* = 20–100 in the HB stretching region. See DOI: 10.1039/c5sc00618j



**DOI:** 10.1039/c5sc00618j

**Published:** 2015-04-14

**Authors:** Sven Heiles, Richard J. Cooper, Matthew J. DiTucci, Evan R. Williams

**Affiliations:** a Department of Chemistry , University of California , B42 Hildebrand Hall , Berkeley , CA 94720 , USA . Email: erw@berkeley.edu

## Abstract

Hydration of gaseous guanidinium (Gdm^+^) with up to 100 water molecules attached was investigated using infrared photodissociation spectroscopy in the hydrogen stretch region between 2900 and 3800 cm^–1^. Comparisons to IR spectra of low-energy computed structures indicate that at small cluster size, water interacts strongly with Gdm^+^ with three inner shell water molecules each accepting two hydrogen bonds from adjacent NH_2_ groups in Gdm^+^. Comparisons to results for tetramethylammonium (TMA^+^) and Na^+^ enable structural information for larger clusters to be obtained. The similarity in the bonded OH region for Gdm(H_2_O)_20_^+^ *vs.* Gdm(H_2_O)_100_^+^ and the similarity in the bonded OH regions between Gdm^+^ and TMA^+^ but not Na^+^ for clusters with <50 water molecules indicate that Gdm^+^ does not significantly affect the hydrogen-bonding network of water molecules at large size. These results indicate that the hydration around Gdm^+^ changes for clusters with more than about eight water molecules to one in which inner shell water molecules only accept a single H-bond from Gdm^+^. More effective H-bonding drives this change in inner-shell water molecule binding to other water molecules. These results show that hydration of Gdm^+^ depends on its local environment, and that Gdm^+^ will interact with water even more strongly in an environment where water is partially excluded, such as the surface of a protein. This enhanced hydration in a limited solvation environment may provide new insights into the effectiveness of Gdm^+^ as a protein denaturant.

## Abstract 2

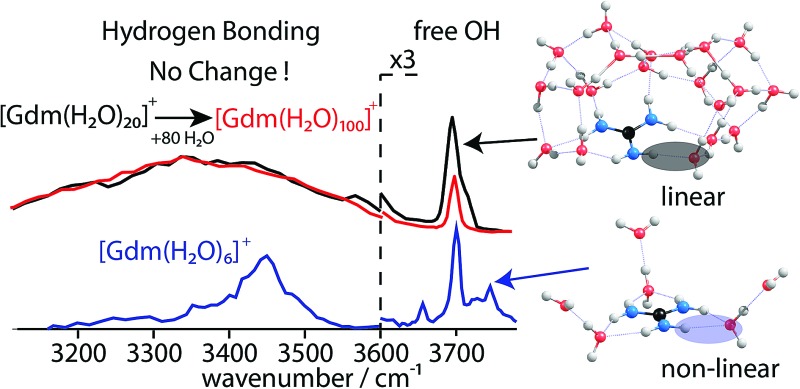
Infrared spectroscopy of guanidinium confined in gaseous nanodrops shows hydration depends on local environment and lends new insights into its effectiveness as a protein denaturant.

## Introduction

Ions are ubiquitous in solution where they play important roles in many different processes in chemistry and biology.^1–7^ Interface phenomena of ions in water, such as the surface activity of ions,^1–3^ or ions in electrochemical processes,^5,6^ have been investigated with a variety of both experimental and computational methods. Arguably, one of the most widely investigated, yet, still hotly debated areas of ion phenomena is the role various salts have on the stabilities of native protein structures.^7,8^ Results from many studies have led to the phenomenological ordering of both anionic and cationic ions based on their effect at either stabilizing or destabilizing the folded structures of proteins, referred to as the “Hofmeister series”, named after the person who first established this ordering over a century ago.^7–9^ A similar ordering of ions occurs for salt solubilities^10,11^ and cloud points of nonionic surfactants.^12^ The effects of ions on protein stability have been attributed to direct ion–protein interactions^8,13–17^ and to ion–water interactions^18–20^ that might affect the hydrogen-bonding network of water molecules that surround proteins.

Of the ions in the Hofmeister series, none is more widely investigated or arguably more important to understand than guanidinium (Gdm^+^).^21–24^ Gdm^+^ is widely used to destabilize or denature proteins in the form of GdmCl or GdmSCN,^14,15,22^ and is often used to rationalize the physical origins of the Hofmeister series.^7,8^ Yet, this ion does not follow the general ordering in charge density observed in the cationic series,^11^ where low charge density ions, such as tetramethylammonium,^25^ stabilize native protein structure, whereas high density ions, such as Mg^2+^ (ref. 10) or Al^3+^ (ref. 26) are destabilizing.

Molecular dynamics (MD) simulations have been used to study various types of ion–protein interactions which could be responsible for the propensity of Gdm^+^ to disrupt the native structure of proteins.^16,27–37^ Various types of Gdm^+^ interactions to proteins have been identified by these studies: the interaction to polar or charged side chain groups,^27–30^ hydrogen bonding to amino acid side chain groups or the peptide backbone^29–32^ and interactions with weakly hydrated, non-polar residues of the protein, *i.e.*, “hydrophobic” interactions to aliphatic^33,37^ or aromatic groups.^16,28,31–33,36^

Although many sites of interaction between Gdm^+^ and various regions of proteins have been identified, there is only limited direct experimental evidence for these types of interactions^13–17,22,23,38–40^ Results from calorimetric measurements indicate a higher local concentration of Gdm^+^ ions at protein surfaces compared to neutral urea molecules, leading the authors to conclude that this difference is due to interactions of Gdm^+^ with negatively charged protein side chains.^17^ Results from base catalysed hydrogen exchange indicate that Gdm^+^ does not interact with the peptide backbone.^38^ Saykally and co-workers recently found evidence for Gdm^+^–Gdm^+^ pairs from X-ray absorption spectroscopy suggesting favourable dispersion interactions between the ions, which could be important to understanding interactions between Gdm^+^ and arginine.^41^ This like-ion pair formation is consistent with some MD simulations.^34^ Results from small angle neutron scattering indicate a preferential interaction of Gdm^+^ with aromatic compared to aliphatic groups.^16^ Wouterson and co-workers found that Gdm^+^ preferentially destabilized β-sheets over α-helices in model proteins which they related to the specific disruption of “hydrophobic” interactions by Gdm^+^.^40^ This interpretation is consistent with the idea that “hydrophobic” interactions promote the formation of β-sheets, whereas they are only of minor importance for the stabilities of α-helices.^42^ Despite the many methods used to study the mechanism of protein structure destabilization by Gdm^+^, the relative contributions of the many different possible interactions of Gdm^+^ with proteins as well as the role of Gdm^+^–water interactions remain a hotly debated topic.

Interactions between Gdm^+^ and water have been investigated,^18,36,43–49^ and some information about how water organizes around Gdm^+^ forming a first solvation shell comes from neutron diffraction studies.^36,45^ The diffraction amplitude from water molecules that are in contact with Gdm^+^ is weak compared to that of many other ions, leading Mason *et al.* to conclude that Gdm^+^ is weakly hydrated.^36,45^ MD simulations show that the neutron scattering data is consistent with linear NH···OH_2_ H-bonds between water molecules in the first hydration shell and Gdm^+^.^36^ These simulations also indicate that the density of water molecules above and below the molecular plane of Gdm^+^ is much lower than that of bulk water indicating that these planes of the ion are “hydrophobic”.^36^ Results from dielectric relaxation spectroscopy indicate that Gdm^+^ salts have a minimal effect on the relaxation time constant of aqueous GdmCl solutions up to ∼7 M when compared to that of pure water, consistent with the concept of weak Gdm^+^ hydration.^44^ Similar conclusions have been drawn from conductivity^46^ and femtosecond IR^47^ measurements. However, a more detailed molecular level understanding of how water interacts with Gdm^+^ remains elusive.

Here, the hydration of Gdm^+^ is investigated by measuring infrared photodissociation (IRPD) spectra of mass selected gaseous clusters that are temperature controlled and trapped in the ion cell of a Fourier-transform ion cyclotron resonance (FT-ICR) mass spectrometer. For clusters with fewer than 10 water molecules, detailed information about Gdm^+^–water interactions are obtained from comparisons to IRPD spectra of computed low-energy structures. For clusters with up to 100 water molecules, comparisons are made to spectra of reference ions that interact weakly or more strongly with water. These results show that hydration of Gdm^+^ is different at small *vs.* large cluster size. Gdm^+^ is weakly hydrated at large cluster size, consistent with previously published reports, but this ion is more strongly hydrated at small cluster size where the arrangement of water molecules differs from that in bulk. These results indicate that the interactions of Gdm^+^ with water depend on its local environment, and these results may shed new light into the effectiveness of Gdm^+^ as a protein denaturant.

## Methods

### Mass spectrometry and IRPD spectroscopy

All experiments were performed using a 7.0 T FT-ICR mass spectrometer, which is based on a 2.75 T FT-ICR instrument that is described elsewhere.^50^ Briefly, hydrated ions are generated by nanoelectrospray ionization (nESI) from 3–5 mM solutions of guanidinium (Gdm^+^), tetramethylammonium (TMA^+^), sodium and cesium chloride salts dissolved in purified water (milli-Q-purification, Millipore, MA, U.S.A.). These solutions are loaded into borosilicate capillaries that have tips that are pulled to an inner diameter of ∼1 μm. A voltage of +650–800 V relative to the heated metal entrance capillary of the mass spectrometer is applied to a platinum filament that is in contact with the sample solution to produce ion-containing nanodrops. The hydrated ions are introduced into the mass spectrometer and are guided into the FT-ICR cell through five stages of differential pumping using electrostatic lenses. A pulse of dry nitrogen gas (∼10^–6^ Torr) is introduced into the vacuum chamber during ion accumulation (∼5 s) to enhance trapping and thermalisation of the ions to the temperature of the surrounding copper jacket. The copper jacket is temperature regulated at 133 K using a controlled flow of liquid nitrogen. After ion accumulation, the pressure returns to <10^–8^ Torr after ∼5 s. Ions of interest are subsequently mass selected by applying a stored waveform inverse Fourier transform waveform. For ions with fewer than 75 water molecules attached, a single precursor ion is isolated, whereas for all larger clusters, an ensemble consisting of three consecutive hydration states is mass selected.

Rate constants for blackbody infrared radiative dissociation (BIRD), which occurs as a result of precursor ions absorbing blackbody photons emitted from the surrounding ion cell and copper jacket, are determined from the precursor and product ion abundances for times between 0.5 and 5.0 s. Infrared photodissociation (IRPD) spectra between 2900 and 3800 cm^–1^ are measured by irradiating the precursor ions with tuneable IR light, which results in increased rates of water molecule loss when the radiation is resonantly absorbed. A spectrum is obtained from the frequency dependent dissociation rate constants corrected for the irradiation time, laser power, and dissociation due to BIRD. Laser light at a repetition rate of 10 Hz is generated by an OPO/OPA system (LaserVision, Bellevue, WA, U.S.A.) pumped by the fundamental (1064 nm) of a Nd:YAG laser (Continuum, Surelight I-19, Santa Clara, CA, U.S.A.). The ion radiation time is chosen to produce significant, but not complete depletion of the precursor ions (typically 0.5–1.0 s) when absorption occurs. A MIDAS data system is used to record the ion signals and all data handling and analysis is done with in-house routines within Matlab 2013a (The MathWorks, Natick, MA, U.S.A.).

### Computational chemistry

Low-energy structures were identified using conformational searches consisting of 1000 individual steps using Macromodel 9.1 (Schrödinger Inc., Portland, OR, U.S.A.) using the OPLS2005 force field. A single search was done for small clusters, whereas up to five conformational searches starting with different initial structures were done for the larger clusters. Between two and five low-energy structures were reoptimized at the B3LYP/6-31++G** level of theory, followed by a harmonic frequency analysis. The water binding energy of H_2_O to Gdm(H_2_O)^+^ was obtained from various low-energy isomeric structures of Gdm(H_2_O)_2_^+^, correcting for the basis set superposition error using the counterpoise method. Q-Chem 4.0 (Q-Chem, Inc., Pittsburgh, PA, U.S.A.)^51^ was used for all quantum chemical computations. Relative Gibbs free energies as a function of temperature were determined from the rotational constants, unscaled harmonic frequencies and electronic ground state energies using an in-house Matlab 2013a (The MathWorks, Natick, MA, U.S.A.) routine.

## Results and discussion

### Evolution of IRPD spectra of Gdm(H_2_O)_*n*_^+^ with cluster size

**Fig. 1:**
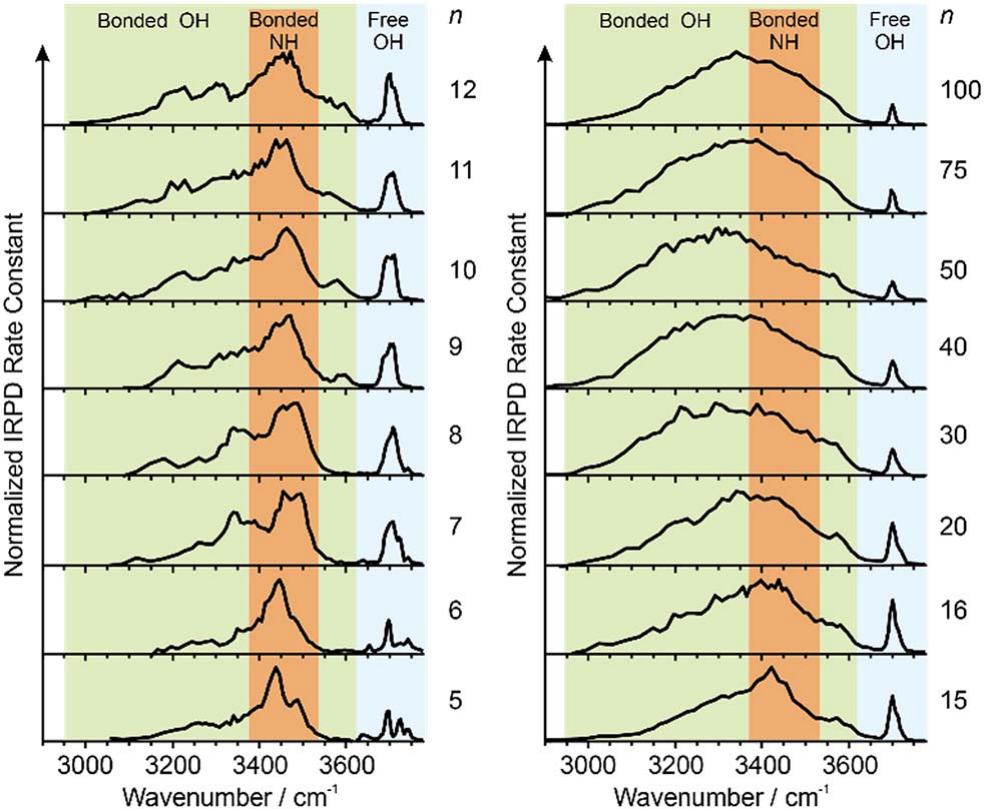
IRPD spectra measured between 2900–3800 cm^–1^ at 133 K for [Gdm(H_2_O)_*n*_]^+^. The free OH (∼3650–3800 cm^–1^), bonded NH_2_ (∼3650–3800 cm^–1^) and bonded OH (∼3650–3800 cm^–1^) regions are shaded in blue, orange and green, respectively.

IRPD spectra of Gdm^+^ with between 5 and 100 water molecules attached in the spectral range between 2900 and 3800 cm^–1^ were measured at 133 K (Fig. 1). The spectra can be divided into three partially overlapping regions. The free OH (fOH) region is between ∼3650 and 3800 cm^–1^ and corresponds to vibrational motions of unperturbed OH bonds of water.^52–60^ Vibrations in this region provide information about the local environment and possible long-range effects of the ion on the hydrogen bonding networks of water molecules at the surface of the cluster.^20^ The two spectral regions from ∼2900–3650 cm^–1^ and ∼3350–3550 cm^–1^ correspond to hydrogen bonded OH and NH_2_ stretches, respectively. Information about the organization of the hydrogen-bonded (HB) water molecules in the ion-containing aqueous nanodrops can be obtained from these bands.^19,53–60^

Comparisons between experimental and calculated spectra of low-energy structures can provide detailed information about ion structure(s), and structural conclusions based on such comparisons for Gdm(H_2_O)_*n*_^+^ with *n* = 1–5 are reported elsewhere.^49^

These results indicate that there are three water molecules in the inner shell which each bind in the interstitial sites and accept hydrogen bonds from adjacent NH_2_ groups, and that additional water molecules form a second solvation shell. Extending these detailed comparisons to much larger clusters is challenging owing to the broad spectral features in the measured spectra and the potentially large number of coexisting and interconverting isomers that are likely present. In order to gain useful information from much larger clusters, comparisons are made to the same size clusters that contain ions for which some structural information is known or can be inferred based on known properties.

### Reference ions

**Fig. 2:**
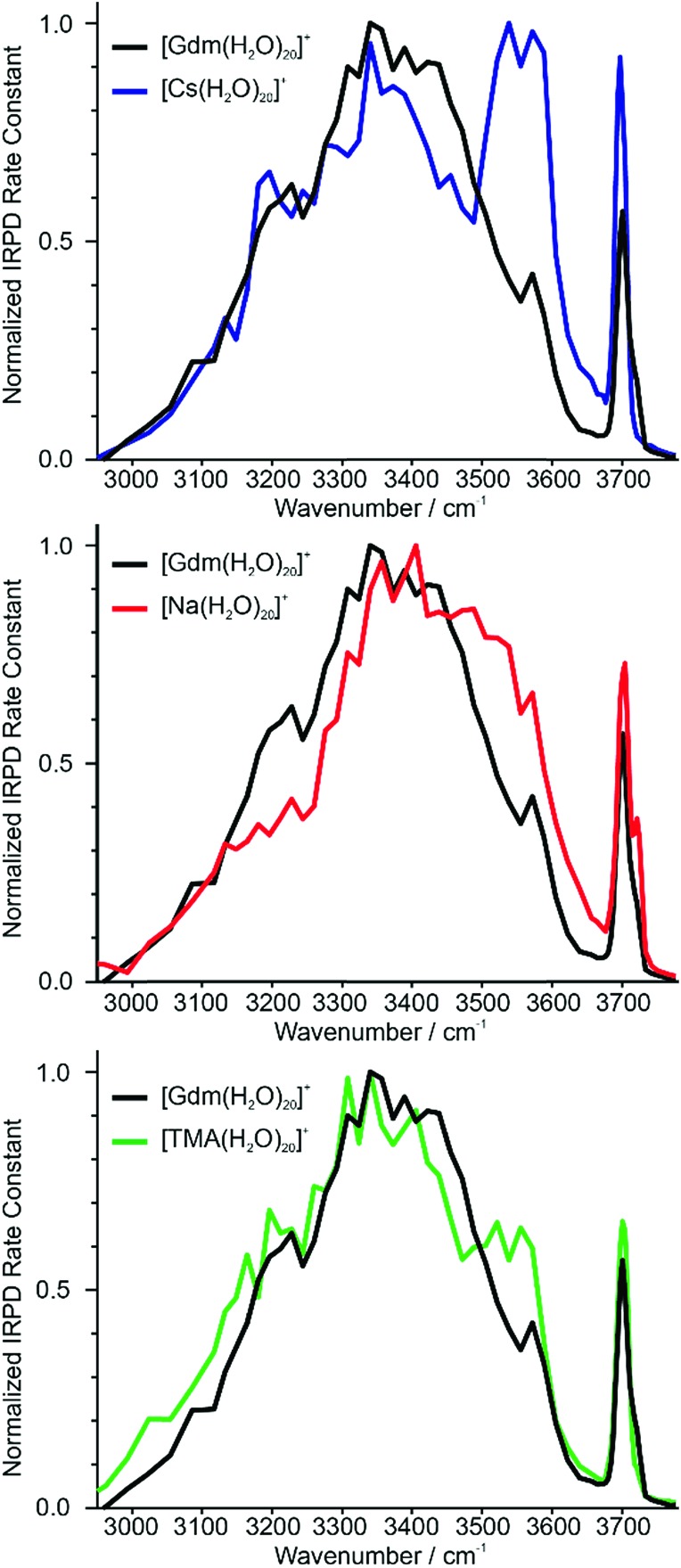
Comparison of IRPD spectra between 2900–3800 cm^–1^ measured at 133 K of [Cs(H_2_O)_20_]^+^ (blue), [Na(H_2_O)_20_]^+^ (red) and [TMA(H_2_O)_20_]^+^ (green) clusters to [Gdm(H_2_O)_20_]^+^ (black).

Identifying suitable reference ions for which information about water organization around the ion is known or can be reasonably surmised can be challenging for a number of reasons. The direct interaction between water molecules in the first hydration shell and the ion can influence the arrangement of water molecules in the subsequent hydration shells,^19,20^ as can the excluded volume (ion size effect)^52^ or the ion charge state.^61^ Ions that can form strong hydrogen bonds to water molecules, such as Gdm^+^ or SO_4_^2–^, can have competing hydration motifs that can potentially further complicate comparisons. Monovalent alkali metal ions are simple, non-reactive ions that do not hydrogen bond to water.^62^ Cs^+^ is the largest nonradioactive ion in this series with an ionic radii of 167 pm,^63^ which is less than the ionic radius of Gdm^+^ in the axial (190 pm)^21^ and radial (230 pm)^21^ directions. Normalized IRPD spectra of Gdm(H_2_O)_20_^+^ and Cs(H_2_O)_20_^+^ are shown in Fig. 2a.

There are two striking features that differentiate the spectra of these clusters. There is only a single band in the fOH region for Cs^+^ indicating a single type of water molecule with a fOH stretch at the surface of the cluster, whereas this band is broader in the spectrum of Gdm^+^, which indicates that there are contributions from a second type of water molecule with a fOH stretch at the cluster surface. The intensity between ∼3500 and 3600 cm^–1^ for Cs^+^ is greater than that for Gdm^+^. The band at ∼3550 cm^–1^ and the relatively sharp fOH band in the spectrum of Cs^+^ is due to a water-clathrate cage around the ion.^64,65^ Such a cage structure also occurs for K(H_2_O)_20_^+^ and Rb(H_2_O)_20_^+^. The extent to which the clathrate structure around these ions affects the hydrogen-bonding network of water at larger cluster sizes is not known.

In contrast to these larger ions, Na^+^ does not induce the formation of a surrounding clathrate,^64^ although it interacts strongly with water. Despite its much smaller size, Na^+^ (102 pm (ref. 63)) was selected as a reference for an ion that is located centrally in the cluster and does not participate in hydrogen bonding to water molecules.^53,64^ The spectrum of Na(H_2_O)_20_^+^ and Gdm(H_2_O)_20_^+^ are compared in Fig. 2b. The spectrum of Na(H_2_O)_20_^+^ has two fOH bands but no prominent feature in the bonded OH region. The broad bonded OH band is blue shifted compared to that in the spectrum of Gdm(H_2_O)_20_^+^.

The other reference ion chosen for comparison is tetramethylammonium (TMA^+^). This ion interacts weakly with water, and although the structures of small water clusters can be affected by the ion charge, the perturbation by the ion to the intrinsic structures of water molecules at larger cluster sizes is minimal making this ion a good choice for essentially ion tagging a neutral water cluster.^66^ TMA^+^ (ionic radius ∼280 pm (ref. 21)) is larger than Gdm^+^. However, this ion is expected to be at the surface of small clusters, and for much larger clusters in which the ion is likely to be at least partially solvated, the excluded volume effect on the overall hydrogen-bonding network of water molecules should be less. Because of the weak ion–water interactions, water molecules more optimally interact with other water molecules in the TMA^+^ containing clusters.^66^

The IRPD spectrum of TMA(H_2_O)_20_^+^ is compared to that of Gdm(H_2_O)_20_^+^ in Fig. 2c. The spectra are remarkably similar in the fOH region. The bonded OH region of TMA(H_2_O)_20_^+^ has a distinct, albeit poorly resolved peak at ∼3550 cm^–1^, but this peak is significantly less pronounced than the corresponding band in the spectrum of Cs(H_2_O)_20_^+^. This suggests that there may be some clathrate-like structure to the water molecules in this cluster.^67^

### Free OH region *n* = 5–16

**Fig. 3:**
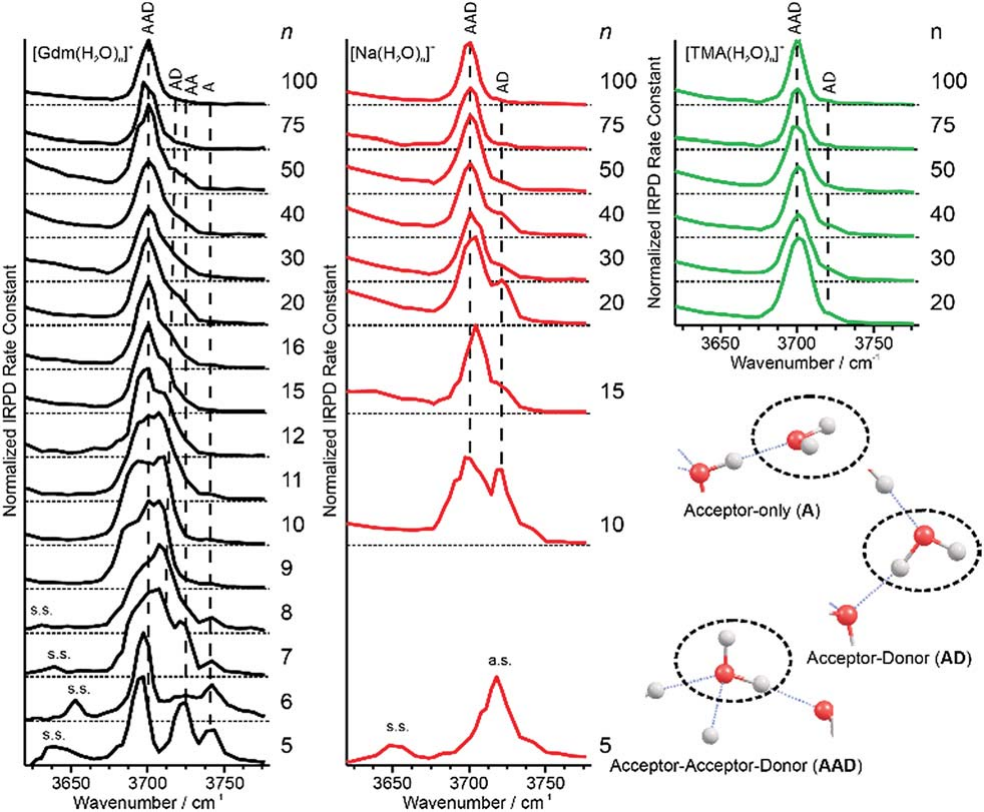
Comparison of the free OH region of Gdm^+^ (black), Na^+^ (red) and TMA^+^ (green) with *n* between 5 and 100 obtained from IRPD measurements at 133 K. Some of the observed vibrations in the free OH region, namely the acceptor-only (**A**), acceptor–donor (**AD**) and acceptor–acceptor–donor (**AAD**) are highlighted in the lower right part of the figure. Oxygen atoms are red and hydrogen atoms are grey.

Information about the structure making or patterning effect of Gdm^+^ on the arrangement of water molecules at the surface of the nanodrop can be inferred from differences in the fOH stretching regions (between 3630 and 3785 cm^–1^) of these ions (Fig. 3).

There are three well resolved and easily identifiable features in the spectrum of Gdm(H_2_O)_5_^+^. The peak at ∼3740 cm^–1^ corresponds to the asymmetric stretch (a.s.) of a water molecule that only accepts a single hydrogen bond (acceptor-only, **A**) and the peaks at ∼3720 and ∼3700 cm^–1^ originate from water molecules that accept two hydrogen bonds (acceptor–acceptor, **AA**) and accept two and donate one hydrogen bond (acceptor–acceptor–donor, **AAD**).^52,55–57,68^ These features show that there are at least three different hydrogen-bonding motifs of water molecules in these small clusters. There is also a peak at ∼3640 cm^–1^, which corresponds to the symmetric stretches (s.s.) of the **A** and **AA** water molecules. These same bands occur in the spectrum of Gdm(H_2_O)_6_^+^ but with different intensities, suggesting similarities in structures of these two clusters (see Fig. S1†). For Gdm(H_2_O)_7_^+^, the **AAD** band is considerably broader indicating the appearance of an additional band with a slightly higher frequency, which is attributed to a water molecule that accepts and donates a single hydrogen bond (acceptor–donor, **AD**). The **A** and **AA** bands diminish in intensity with increasing cluster size, and these bands are essentially absent in the spectra of clusters with *n* ≥ 9. A band at ∼3580 cm^–1^ appears in the spectrum of Gdm(H_2_O)_9_^+^ (Fig. 1), which indicates the presence of water molecules without free OH bands. For larger clusters, the fOH region consists of only broad overlapping **AD** and **AAD** bands and the relative intensity of the **AAD** stretch increases with cluster size. The **AD** stretch is no longer a distinct band but leads to a broadening of the **AAD** band towards higher energies for clusters with *n* ≥ 16.

The fOH bands in the spectra of Na(H_2_O)_*n*_^+^, *n* = 5, 10 and 15 differ significantly from those in the corresponding spectra of Gdm^+^ (Fig. 3). The spectrum of Na(H_2_O)_5_^+^ has just a single symmetric stretch at ∼3650 cm^–1^ and the corresponding asymmetric stretch at ∼3740 cm^–1^, which is in good agreement with the previously reported spectrum.^53^ For Na(H_2_O)_10_^+^, there are two bands corresponding to **AD** and **AAD** water molecules, which are well resolved compared to these features in the spectrum of Gdm(H_2_O)_10_^+^. This indicates that most of the **AD** and **AAD** oscillators in this sodium containing cluster experience a similar hydrogen bonding environment, whereas the environment is more heterogeneous for these oscillators in the corresponding Gdm^+^ cluster. For clusters with 15 water molecules, the ratio of the **AAD** to **AD** band is significantly higher for Na^+^ than for Gdm^+^, indicating that surface water molecules reside in a more ordered environment for the former ion.

### Structures of small Gdm^+^ and Na^+^ clusters

**Fig. 4:**
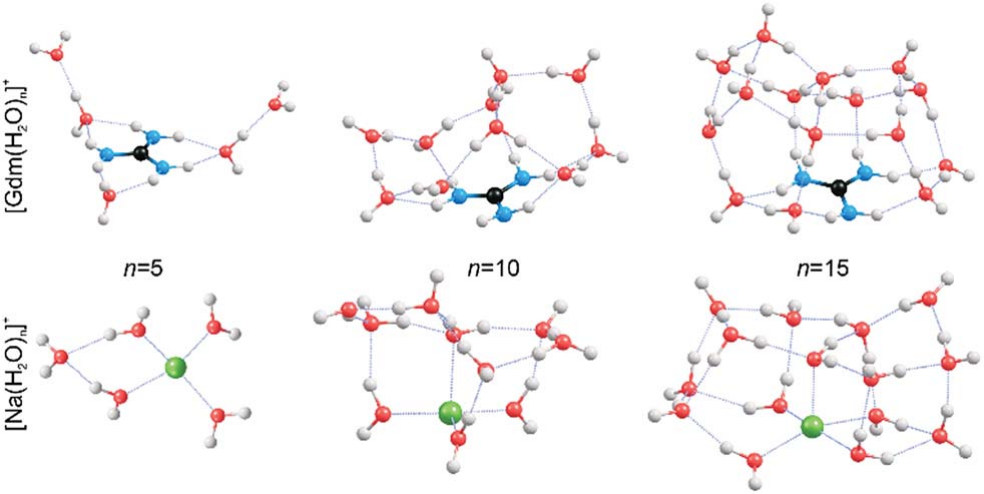
Representative structures of [Gdm(H_2_O)_*n*_]^+^ and [Na(H_2_O)_*n*_]^+^ obtained from B3LYP/6-31++G** calculations. Oxygen, hydrogen, carbon, nitrogen and sodium atoms are shown as red, white, black, blue and green spheres, respectively.

In order to obtain qualitative information from the IRPD spectra, low-energy isomers at select cluster sizes were identified computationally and these structures are shown in Fig. 4.

Structures for these ions with five water molecules were reported previously,^49,53^ but the low-energy structures for the larger clusters may not be global minimum structures owing to the complex conformational space and modest levels of theory used. It is almost certain that multiple isomers contribute to the IRPD spectra at the larger cluster sizes.

Consistent with results from experiment, the calculated structures indicate that the hydration of Gdm^+^ and Na^+^ differs significantly. For Na(H_2_O)_5_^+^, the first hydration shell is complete with four water molecules that are evenly dispersed around the central Na^+^, and the fifth water molecule occupies a second shell in which it accepts hydrogen bonds from two inner shell water molecules. In contrast, the first solvent shell is complete with three water molecules for Gdm(H_2_O)_5_^+^ and these water molecules only interact with the NH_2_ groups. The two additional water molecules occupy a second solvation shell and accept one hydrogen bond from an inner shell water molecule. In contrast to the more spherical hydration of Na^+^, the hydration of Gdm^+^ occurs roughly in the plane of the molecule. No water molecules attach to the central carbon atom despite its partial positive charge.^36,49^

Comparisons between experimental and computed spectra of candidate low-energy structures for clusters with *n* = 6–9 were performed (Fig. S1–S5†). For Gdm(H_2_O)_6_^+^, the structure corresponds to one in which the core structure established for the smaller clusters continues such that each of the three interstitial water molecules in the inner shell form a single hydrogen bond to an outer shell water molecule (Fig. S1†). With *n* ≥ 7, the onset of water bridges between the three distinct hydration sites of the first shell consisting of **AD** water molecules occurs, which broadens the band at 3700 cm^–1^ (see Fig. S2–5†). This occurs until *n* = 9 where the absence of an **A** or **AA** band indicates that all water molecules donate and accept at least one hydrogen bond.

The absence of the **A** and **AA** stretches in the IRPD spectra at *n* ≥ 9 indicates a change in the inner shell hydration of Gdm^+^ occurs. The orientation of water molecules that coordinate to two adjacent NH_2_ groups results in a separation of the second shell water molecules from each other by at least 870 pm (O–O distance), which prevents hydrogen bonding interactions between water molecules in the second solvation shell. In order for every water molecule to donate and accept at least one hydrogen bond, the water molecules in the first solvation shell must rearrange by accepting a hydrogen bond from just one NH_2_ group. This rearrangement of shell structure is consistent with the appearance of the band at 3580 cm^–1^ (Fig. 1) attributed to **ADD** water molecules.^54,64^

The structural rearrangement of the inner solvation shell that starts to occur for *n* ≥ 9 inferred from the IRPD spectra is also found in the computed structures (Fig. 4 and S4–S5†). For Gdm(H_2_O)_10_^+^, there are no water molecules that occupy the interstitial sites in which one water molecule accepts hydrogen bonds from two NH_2_ groups (Fig. 4). Instead, water molecules only accept a single H-bond from Gdm^+^. This arrangement of water around the central ion is favourable for optimizing the number of water–water hydrogen bonds. The linear NH_2_···OH_2_ coordination pattern continues for larger clusters (Fig. 4) where water molecules start to form a dome-like structure above the central carbon atom of Gdm^+^. The broad OH feature for these clusters is consistent with the anisotropic environment of the water molecules in the Gdm^+^ clusters in this size range.

The hydration is significantly different for the small sodium clusters where the second shell water molecule in Na(H_2_O)_5_^+^ accepts hydrogen bonds from two inner shell water molecules, and subsequent water molecules can readily form two or more hydrogen bonds to other water molecules resulting in quasi-spherical solvation. The number of different water molecules, estimated by their local hydrogen bond environments, is fewer in the intermediate size clusters of Na^+^ compared to that for Gdm^+^. This is consistent with the better resolved **AD** peaks in the spectra of Na^+^ for *n* = 10 and 15 compared to that for Gdm^+^. The lack of water interactions with the central carbon of Gdm^+^ results in quasi-planar growth of the cluster for *n* ≤ 8, whereas Na^+^ clusters undergo quasi-spherical growth. This results in a different arrangement of water molecules at the surface and differences in the fOH region of the IRPD spectra of these two ions at small cluster size.

### Energetics of the first hydration shell rearrangement

In order to gain insight into why the coordination number of Gdm^+^ changes with cluster size, the binding energies of a water molecule to Gdm^+^(H_2_O) at different sites in Gdm(H_2_O)_2_^+^ were calculated. The binding energy of a water molecule that forms a single hydrogen bond to the inner shell water molecule is 69 kJ mol^–1^. If the water molecule occupies an interstitial site and accepts two hydrogen bonds, one from each adjacent NH_2_, the binding energy is 80 kJ mol^–1^. Thus, each H-bond in this configuration is 40 kJ mol^–1^. If instead the water is in the plane of Gdm^+^ with an N–H···O angle of 180° to a NH_2_ group forming only a single hydrogen bond (the N–H···O bond lengths were optimized while the other coordinates were constant), then the binding energy is 65 kJ mol^–1^.

Thus, the most energetic configuration for water is to bind between two adjacent NH_2_ groups at small cluster size, but the sum of energies of two linear H-bonds with two different water molecules is greater than that of the two non-optimal H-bonds of a single water molecule occupying the interstitial site. After these interstitial sites are occupied by the first three water molecules, additional outer shell water molecules will have only a single hydrogen bond. As more outer shell water molecules are added, there is a driving force to gain water–water hydrogen bonds by pulling the inner shell water molecules from the interstitial sites so that there is more overall hydrogen bonding.

### Evidence for spherical clusters at large size

Extrapolation of cluster data to obtain bulk phase properties has been done to obtain absolute electrochemical potentials,^69^ formation enthalpies of metal clusters^70^ and the electron hydration enthalpy.^71^ The surface-to-volume ratio of a sphere changes as *n* ^–1/3^. The IRPD measurements clearly distinguish water molecules at the cluster surface with a fOH stretch from water molecules that have both hydrogen atoms involved in hydrogen bonds that are located in the interior or at the surface. For small clusters, effects of transition dipole moments and binding energies of individual water molecules must be known in order to evaluate experimental intensities. For sufficiently large clusters where these values should not depend strongly on cluster size, the ratio of the integrated fOH and the integrated HB band should be roughly linear with *n* ^–1/3^ and extrapolate to zero at infinitely large cluster size.

**Fig. 5:**
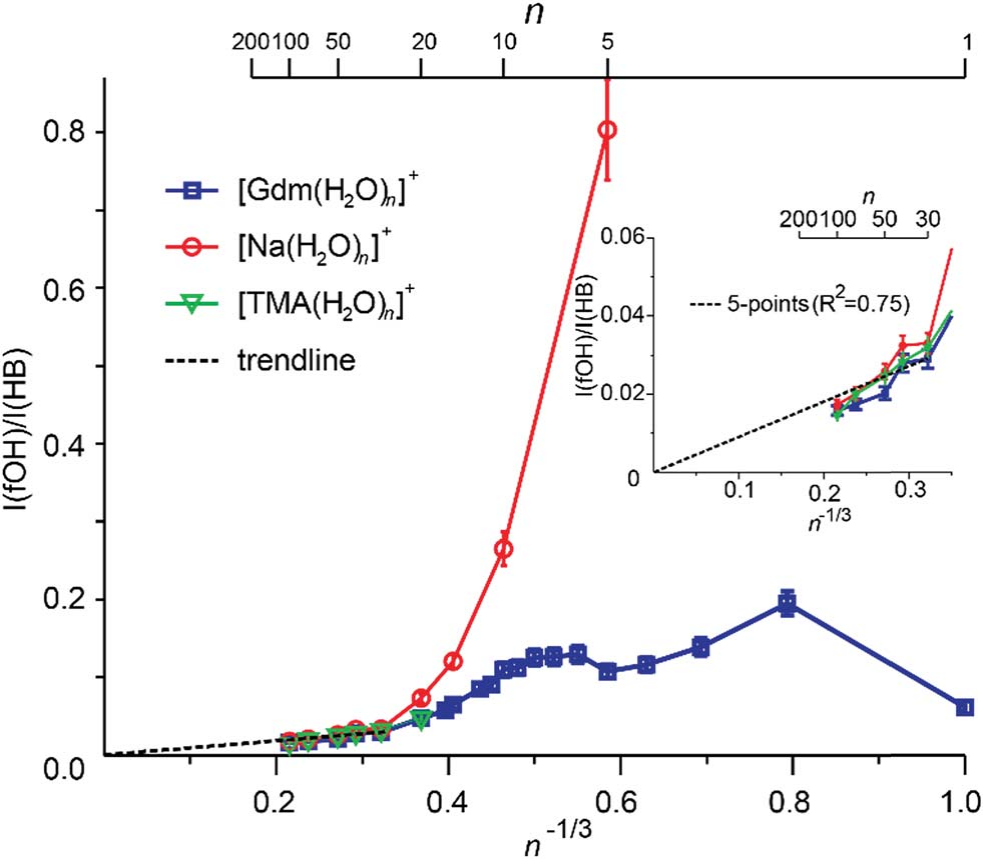
The ratio for the integrated fOH to HB intensities (*I*(fOH)/*I*(HB)) obtained by numerical integration of the experimental IRPD intensities of Gdm^+^ (blue), Na^+^ (red) and TMA^+^ (green) water drops as a function of *n* ^–1/3^. The inset shows an expansion for the clusters with *n* = 30–100 and linear fit (black dashed line) to the five largest clusters.

A plot of the ratio of fOH to HB intensities (*I*(fOH)/*I*(HB)) as a function of *n* ^–1/3^ obtained by integrating the IRPD intensities from 3650–3800 cm^–1^ and 2900–3650 cm^–1^, respectively, for Gdm^+^, Na^+^ and TMA^+^ are shown in Fig. 5.

The uncertainties are obtained from four measurements of Gdm(H_2_O)_100_^+^ (see Fig. S7†) and these same relative uncertainties were used for all other cluster sizes. For Na^+^, the *I*(fOH)/*I*(HB) is highest for *n* = 5 where the first HB band is observed, and decreases rapidly with cluster size until *n* ∼ 20. From *n* = 20 to 100, these data are linear and extrapolate to zero at infinite cluster size. Similar results are observed for TMA^+^.

In contrast, the *I*(fOH)/*I*(HB) values for Gdm^+^ are much lower than those for Na^+^ at small cluster size. Gdm^+^ differs from Na^+^ in that the inner shell water molecules hydrogen bond directly to the ion and these contribute to the bonded OH region of Gdm^+^ at these smaller cluster sizes. A clear decrease in *I*(fOH)/*I*(HB) is not observed for Gdm^+^ until *n* ∼ 12, consistent with anisotropic solvation of Gdm^+^ at smaller cluster size.

The *I*(fOH)/*I*(HB) values for all three ions are indistinguishable within the accuracy of these measurements for *n* ≥ 20. Results for a linear fit (*R* ^2^ = 0.75) of these data for these larger clusters for all three ions is inset in Fig. 5. Both the linearity of these data and the *y*-intercept of zero indicate that the number of water molecules on the surface of the water nanodrops relative to those in the interior does not depend significantly on the identity of the cation for *n* ≥ 20 and that these larger clusters are spherical to a large extent.

### Spectra of clusters with between 20 and 100 water molecules

**Fig. 6:**
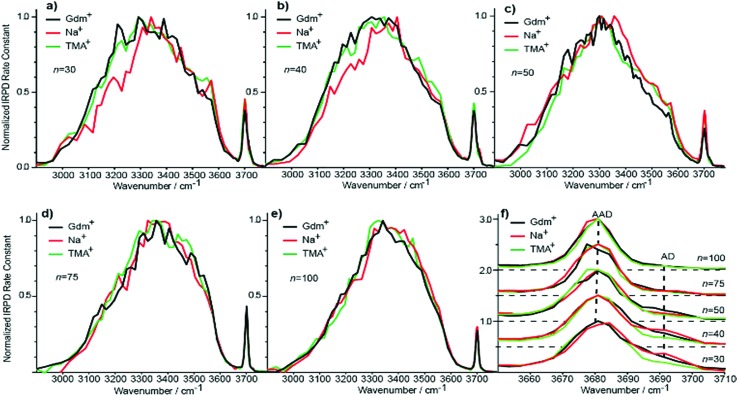
Comparison of IRPD spectra between 2900–3800 cm^–1^ measured at 133 K for Gdm^+^ (black), Na^+^ (red) and TMA^+^ (green) with (a) 30, (b) 40, (c) 50, (d) 75 and (e) 100 water molecules. The fOH stretching region of all cluster sizes with the corresponding highlighted AD and AAD stretches is expanded in (f).

Although the *I*(fOH)/*I*(HB) values for the three ions are indistinguishable for *n* ≥ 20, distinguishing spectral features persist for some of these larger clusters. The fOH region of the three ions are distinctly different at *n* = 20, where the intensity of the **AD** band relative to the **AAD** band follows the order Na^+^ > Gdm^+^ > TMA^+^. This indicates water molecules at the surface of the clusters are more optimally hydrogen bonded for TMA^+^ than for Na^+^ and Gdm^+^ is intermediate. This general trend persists for *n* = 30 and 40 (Fig. 6f). The **AD**/**AAD** ratio becomes small and is indistinguishable for the three ions for *n* between 50 and 100. This indicates that the effect of the individual ions on the hydrogen bonding network of the water molecules at the surface of these clusters is negligible for *n* ≥ 50.

There are also differences in the HB region for these different ions with *n* ≥ 20, and the IRPD spectra of Gdm^+^, Na^+^ and TMA^+^ for *n* = 30 and 40 are shown in Fig. 6a and b. The HB region for Gdm^+^ and TMA^+^ consists of a single broad band with a maximum around 3300–3400 cm^–1^. In contrast, this band for Na^+^ is asymmetrical with lower intensity between 3100 and 3300 cm^–1^ at both cluster sizes consistent with bulk solution measurements.^72^ For *n* = 50, 75 and 100, the spectra of these ions are similar (Fig. 6c–e). The spectrum of Na(H_2_O)_50_^+^ has slightly higher intensity on the blue edge of the HB band compared to the other ions, but the intensity at the lower frequencies is nearly the same. The HB band for TMA^+^ is much more similar to that of Gdm^+^ than Na^+^ and this difference is significant for clusters up to *n* = 50. This is also supported by quantitatively comparing the HB band of Gdm^+^, TMA^+^ and Na^+^ as presented in Table S1.† These results show that the HB network of water molecules solvating Gdm^+^ is structurally more similar to the HB network of TMA^+^ hydrates than Na^+^ hydrates. This indicates that Na^+^ perturbs the HB network of water more than the other two ions, and that this perturbation of water structure propagates to the surface of nanodrops containing up to 50 water molecules.

In order to visualize possible effects of these ions on water structure, some representative low-energy structures were identified for these ions at *n* = 20 and 40 (Fig. S8†). As was the case for Gdm(H_2_O)_10_^+^, water molecules only accept single hydrogen bonds from the central ion and the central carbon atom does not interact with water resulting in a cavity above the plane of the ion. This is consistent with both experimental and theoretical results for Gdm^+^ in water.^36,44–46^ TMA^+^ cannot form H-bonds with water, which results in a similar exclusion zone as that above the plane of Gdm^+^. In contrast, Na^+^ binds water strongly in the first solvation shell and this changes the optimal bond length to other water molecules (Fig. S8†).

### Comparison of Gdm^+^ and Na^+^ with 20 and 100 water molecules

**Fig. 7:**
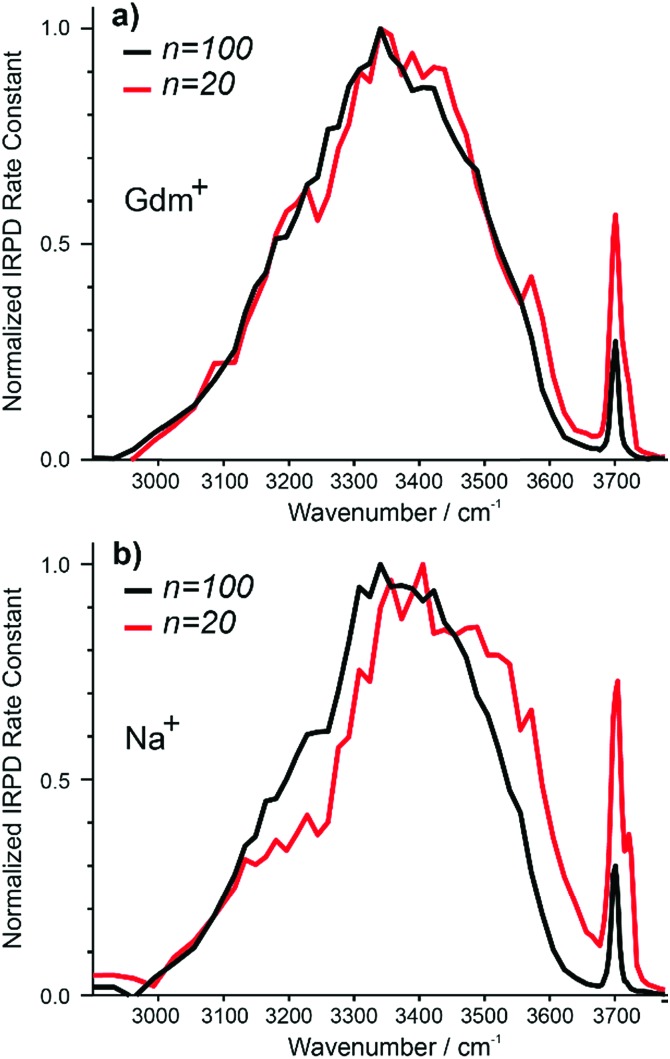
Comparison of IRPD spectra between 2900–3800 cm^–1^ measured at 133 K for Gdm^+^ (a) [Gdm(H_2_O)_100_]^+^ (black), [Gdm(H_2_O)_20_]^+^ (red) as well as (b) [Na(H_2_O)_100_]^+^ (black), [Na(H_2_O)_20_]^+^ (red).

The IRPD spectra of Gdm^+^ with 20 and 100 water molecules and the corresponding spectra for Na^+^ are shown in Fig. 7a and b. For Gdm^+^, the spectra differ most significantly in the relative intensities of the fOH and HB bands. For *n* = 100, the relative contribution of the fOH is lower owing to the smaller surface-to-volume ratio for the larger cluster. The fOH peak is also narrower consistent with a significantly lower contribution of **AD** water molecules at the surface. This indicates that water at the surface of the larger cluster is more homogenous, whereas that in the smaller cluster is more disordered.

However, the intensity and appearance of the HB region is remarkably similar for these two cluster sizes indicating that the HB network between water molecules is similar.

In contrast, the differences in the spectra of Na^+^ with 20 and 100 water molecules are more striking, both in the fOH and HB regions. More disorder at the surface of the smaller cluster is also indicated by the significant **AD** stretch, but the HB region for Na (H_2_O)_20_^+^ has more intensity around 3500–3600 cm^–1^ and less intensity between 3100–3300 cm^–1^ compared to that for Na (H_2_O)_100_^+^. These results indicate that the HB network of water molecules in the Na^+^ clusters change significantly between *n* = 20 and 100, whereas there is only a minor change in the network of water molecules for Gdm^+^ over this same size range.

## Conclusion

The IRPD spectra of guanidinium hydrated with up to 100 water molecules along with spectra of corresponding hydrated ions of sodium and tetramethylammonium provide new insights into the unique way in which Gdm^+^ interacts with water. For small clusters of Gdm^+^ (*n* < 9), a detailed comparison of the IRPD spectra to spectra computed from low-energy structures indicates that Gdm^+^ interacts strongly with water in the plane of the ion *via* H-bonding in which three water molecules that form an inner shell each accept H-bonds from adjacent NH_2_ groups and subsequent water molecules form a second solvation shell by hydrogen bonding to an inner shell water molecule. This near-planar solvation continues until *n* > 8 where more optimal water–water H-bonding results in an expansion of the inner shell in which water molecules form only a singly linear H-bond to the ion in order to optimize the number of water–water hydrogen bonds. Water forms a “dome” over the central carbon for even larger clusters despite the partial positive charge on the carbon atom as a result of little orbital density around this atom.^49^

The similar appearance of the HB region of Gdm^+^ with 20 and 100 water molecules and the similar appearance of the HB region between similar size clusters of Gdm^+^ and TMA^+^ both indicate that Gdm^+^ has a minimal effect on the hydrogen-bonding network of water molecules for these larger cluster sizes. Yet, TMA^+^ and Gdm^+^ are on opposite ends of the Hofmeister ion series and affect the stabilities of native proteins differently. The relatively weak interaction of both Gdm^+^ and TMA^+^ with water has led some to conclude that ion–water interactions do not play a significant role in the Hofmeister behaviour of ions. However, the way in which Gdm^+^ interacts with water depends both on the cluster size and the orientation of water with respect to the ion. Gdm^+^ effectively hydrogen bonds with water in the plane of the ion, but not above and below the plane where water–water hydrogen bonding is favoured. Enhanced water–water hydrogen bonds within the plane also results in a different orientation and H-bonding motif of water to the ion when there are a sufficient number of water molecules. The way in which Gdm^+^ interacts with water will thus depend on its local environment. Gdm^+^ will interact with water in the plane of the ion even more strongly in an environment where water is excluded, such as the surface of a protein, and the hydrophobic nature of the ion above and below the plane should enhance such interactions with hydrophobic regions. Both the asymmetric hydration behaviour of Gdm^+^^36,45,49^ as well as its enhanced interactions with water in a limited solvated environment, may explain its effectiveness as a protein denaturant. These surfactant-like properties may stabilize hydrophobic regions of the protein in water and the enhanced interactions with water in a limited solvation environment should lower barriers to protein unfolding. This suggests that the mechanism by which Gdm^+^ affects the stabilities of folded proteins is fundamentally different than that of many other ions, such as the sulfate dianion, where long-range effects of the ion on the hydrogen-bonding network of water molecules has been observed, both in gaseous clusters^20^ as well as to a more limited extent in the condensed phase.^73^ GdmCl destabilizes protein structure, whereas Gdm_2_SO_4_ slightly stabilizes protein structure;^22,43^ this opposite effect with these two anions may be related to their very different interactions with water and effects on the water–water hydrogen bonding network. Similar investigations into other ions in the Hofmeister series may lead to additional insights into these phenomena.
