# Supplementary material for: Hydration of guanidinium depends on its local environment
Source: Chem Sci. 2015 Apr 14;6(6):3420–9. doi: 10.1039/c5sc00618j (PMC5490459; doi:10.1039/c5sc00618j)
Supplement: Supplementary file 1 [file SC-006-C5SC00618J-s001.pdf]

Electronic Supplementary Material (ESI) for Chemical Science.
This journal is © The Royal Society of Chemistry 2015

Hydration of Guanidinium Depends on Its Local Environment

Sven Heiles[§], Richard J. Cooper, Matthew J. DiTucci and Evan R. Williams*

Department of Chemistry, University of California, Berkeley, California 94720-1460

Supporting Information

Full Citation for Reference 51 :

[51] Shao, Y.; Molnar, L. F.; Jung, Y.; Kussmann, J.; Ochsenfeld, C.; Brown, S. T.; Gilbert, A. T. B.; Slipchenko, L. V.; Levchenko, S. V.; O'Neill, D. P.; DiStasio, R. A.; Lochan, R. C.; Wang, T.; Beran, G. J. O.; Besley, N. A.; Herbert, J. M.; Lin, C. Y.; Van Voorhis, T.; Chien, S. H.; Sodt, A.; Steele, R. P.; Rassolov, V. A.; Maslen, P. E.; Korambath, P. P.; Adamson, R. D.; Austin, B.; Baker, J.; Byrd, E. F. C.; Dachsel, H.; Doerksen, R. J.; Dreuw, A.; Dunietz, B. D.; Dutoi, A. D.; Furlani, T. R.; Gwaltney, S. R.; Heyden, A.; Hirata, S.; Hsu, C. P.; Kedziora, G.; Khalliulin, R. Z.; Klunzinger, P.; Lee, A. M.; Lee, M. S.; Liang, W.; Lotan, I.; Nair, N.; Peters, B.; Proynov, E. I.; Pieniazek, P. A.; Rhee, Y. M.; Ritchie, J.; Rosta, E.; Sherrill, C. D.; Simmonett, A. C.; Subotnik, J. E.; Woodcock, H. L.; Zhang, W.; Bell, A. T.; Chakraborty, A. K.; Chipman, D. M.; Keil, F. J.; Warshel, A.; Hehre, W. J.; Schaefer, H. F.; Kong, J.; Krylov, A. I.; Gill, P. M. W.; Head-Gordon, M. Advances in Methods and Algorithms in a Modern Quantum Chemistry Program Package. *Phys. Chem. Chem. Phys.* **2006**, *8*, 3172-3191.

Comparison of the Experimental and Theoretical IRPD spectra for $[Gdm(H_2O)_n]^+$ with $n = 6 - 9$

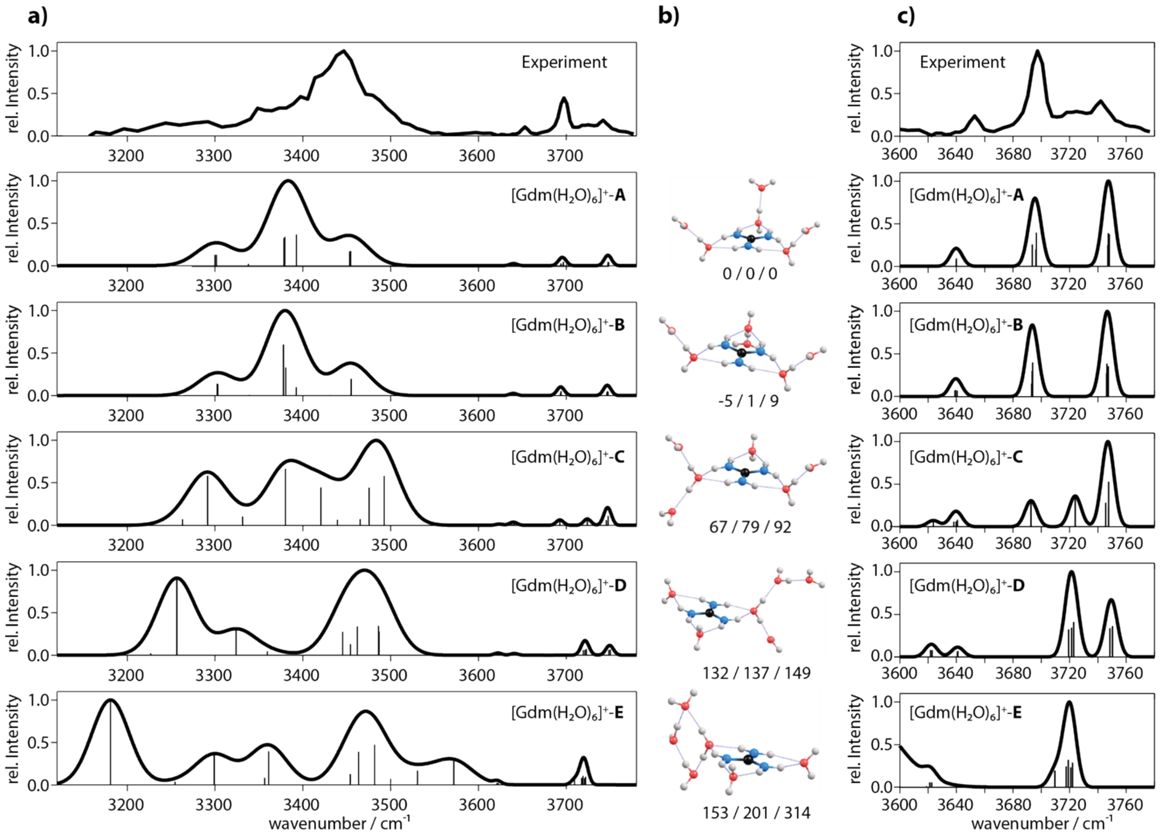

Figure S1. Comparison of the normalized (a) full and (c) free OH region (3620−3780 cm^{-1}) of the experimental IRPD spectrum of $[Gdm(H_2O)_6]^+$ at 133 K (upper panel) to the calculated harmonic IR spectra (lower panels) of the corresponding structures shown in (b). All structures and frequency calculations were performed at the B3LYP/6-31++G** level of theory, a frequency scaling factor of 0.954 to account for anharmonic corrections was used and the frequencies in the free and bonded OH region were convoluted with Gaussians with a width of 15 and 60 cm^{-1}, respectively. The Gibbs Enthalpies at 0, 133 and 300 K in meV are given below each isomer in (b) relative to isomer A.

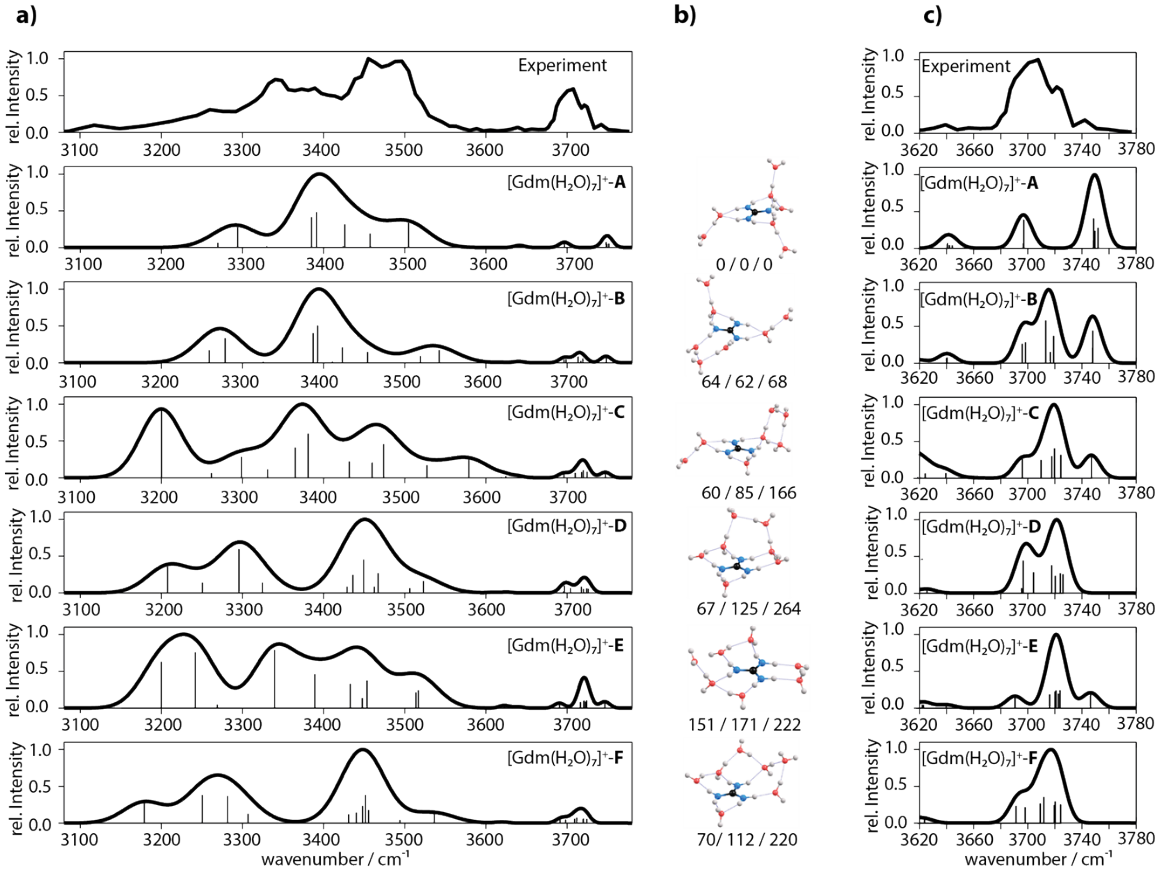

Figure S2. Comparison of the normalized (a) full and (c) free OH region (3620−3780 cm^{-1}) of the experimental IRPD spectrum of $[Gdm(H_2O)_7]^+$ at 133 K (upper panel) to the calculated harmonic IR spectra (lower panels) of the corresponding structures shown in (b). All structures and frequency calculations were performed at the B3LYP/6-31++G** level of theory, a frequency scaling factor of 0.954 to account for anharmonic corrections was used and the frequencies in the free and bonded OH region were convoluted with Gaussians with a width of 15 and 60 cm^{-1}, respectively. The Gibbs Enthalpies at 0, 133 and 300 K in meV are given below each isomer in (b) relative to isomer A.

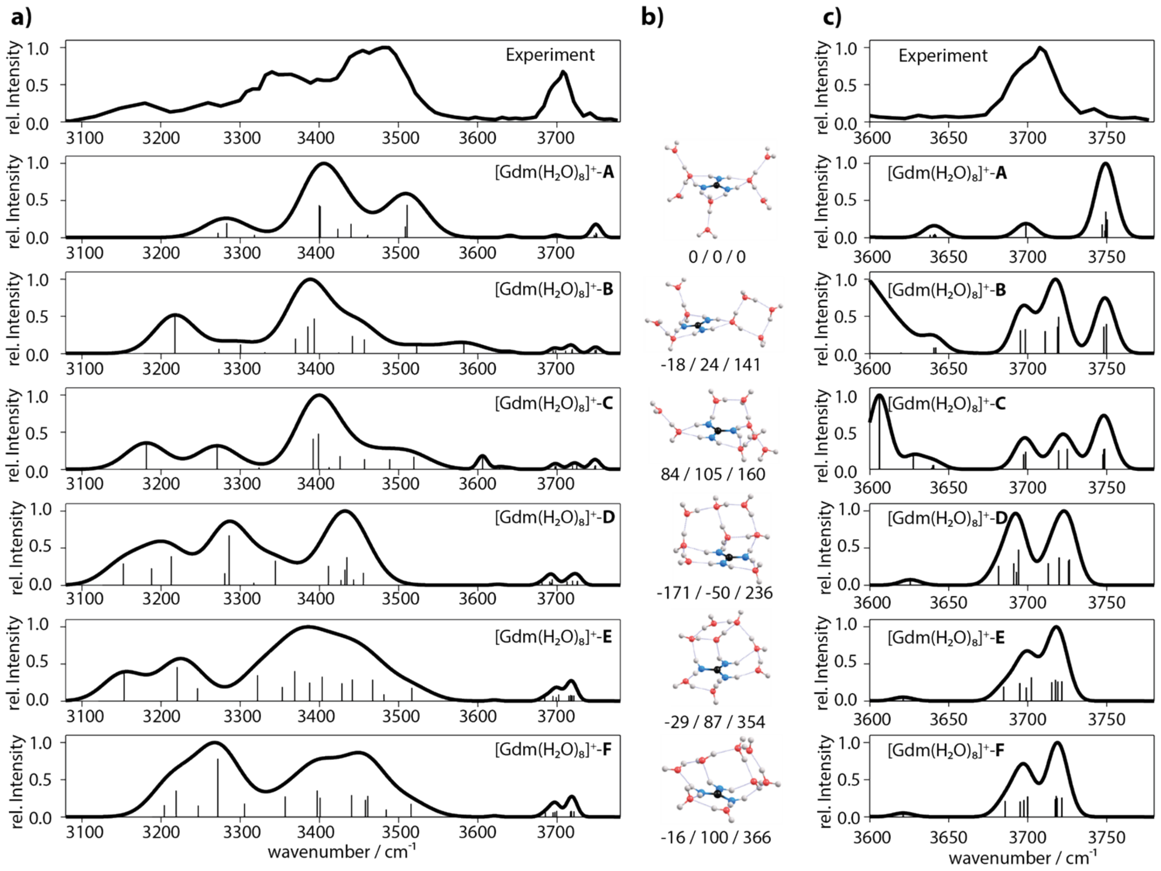

Figure S3. Comparison of the normalized (a) full and (c) free OH region (3620−3780 cm^{-1}) of the experimental IRPD spectrum of $[Gdm(H_2O)_8]^+$ at 133 K (upper panel) to the calculated harmonic IR spectra (lower panels) of the corresponding structures shown in (b). All structures and frequency calculations were performed at the B3LYP/6-31++G** level of theory, a frequency scaling factor of 0.954 to account for anharmonic corrections was used and the frequencies in the free and bonded OH region were convoluted with Gaussians with a width of 15 and 60 cm^{-1}, respectively. The Gibbs Enthalpies at 0, 133 and 300 K in meV are given below each isomer in (b) relative to isomer A.

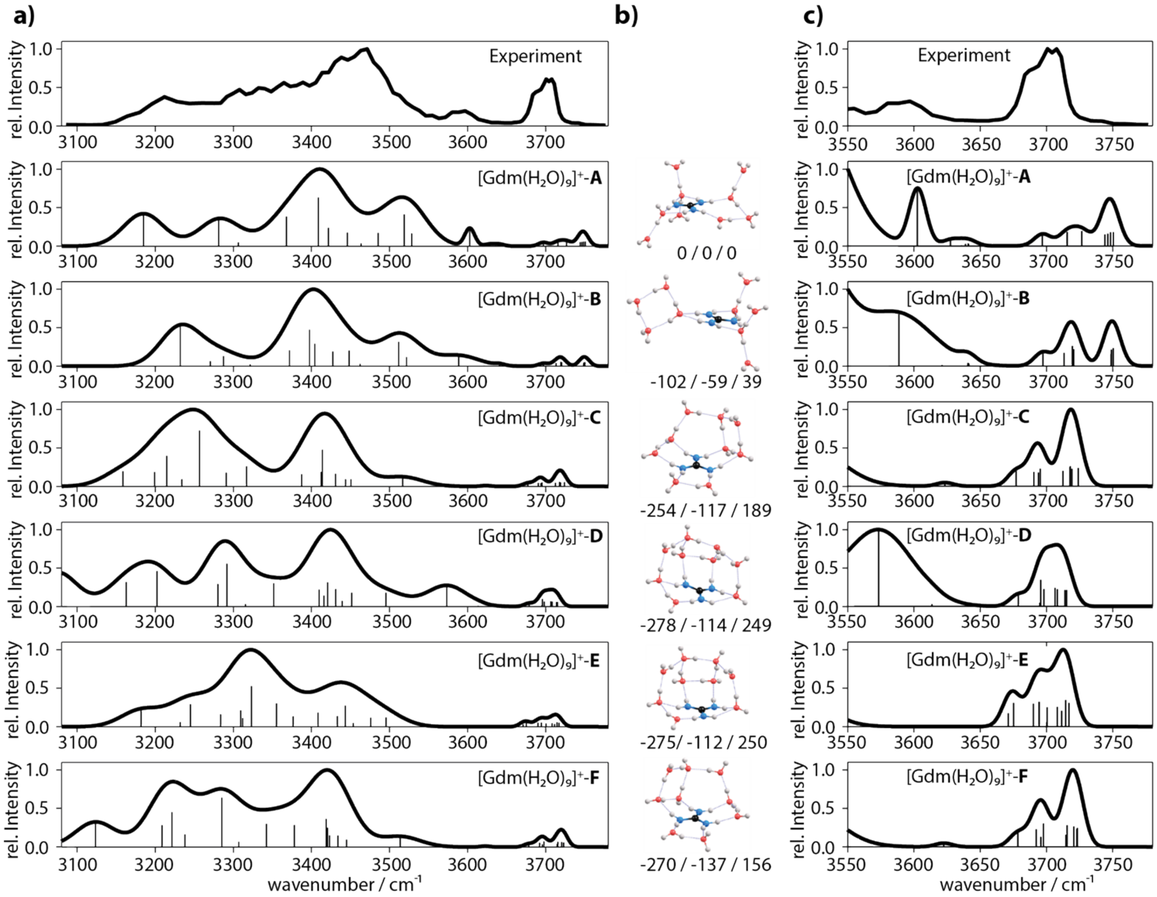

Figure S4. Comparison of the normalized (a) full and (c) free OH region (3620−3780 cm^{-1}) of the experimental IRPD spectrum of $[Gdm(H_2O)_9]^+$ at 133 K (upper panel) to the calculated harmonic IR spectra (lower panels) of the corresponding structures shown in (b). All structures and frequency calculations were performed at the B3LYP/6-31++G** level of theory, a frequency scaling factor of 0.954 to account for anharmonic corrections was used and the frequencies in the free and bonded OH region were convoluted with Gaussians with a width of 15 and 60 cm^{-1}, respectively. The Gibbs Enthalpies at 0, 133 and 300 K in meV are given below each isomer in (b) relative to isomer A.

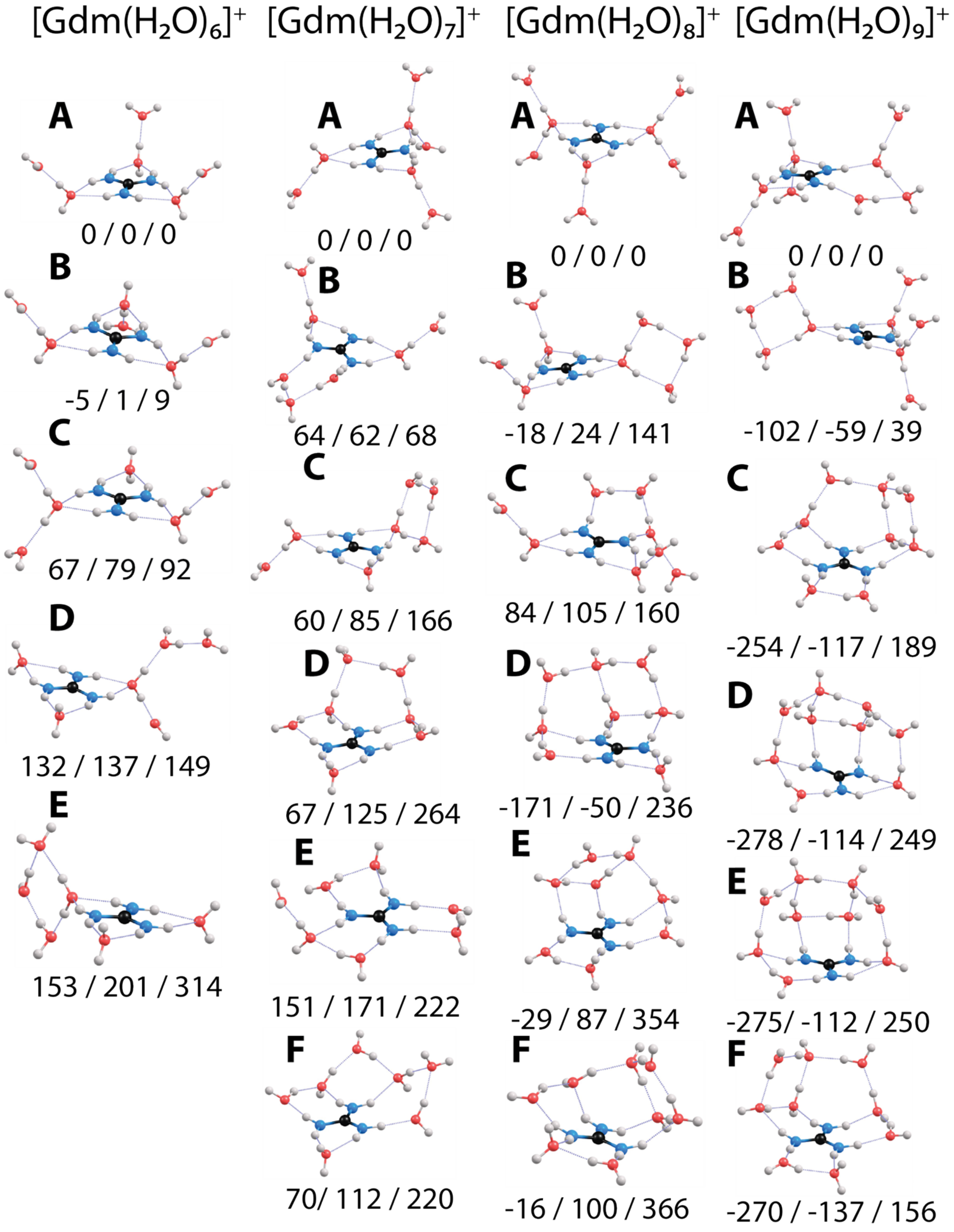

Figure S5. Low-energy isomers for $[Gdm(H_2O)_6]^+$, $[Gdm(H_2O)_7]^+$, $[Gdm(H_2O)_8]^+$ and

$[Gdm(H_2O)_9]^+$ (B3LYP/6-31++G**). The Gibbs Enthalpies at 0, 133 and 300 K in meV are given below each isomer relative to isomer A.

Experimental details and reproducibility

All experimental spectra for $[Gdm(H_2O)_n]^+$, $[Na(H_2O)_n]^+$, $[Cs(H_2O)_n]^+$, and $[TMA(H_2O)_n]^+$, with the same number of water molecules *n* attached where measured within 24 hours to ensure comparability of the spectra. BIRD rate constants were remeasured after 5 to 15 IRPD data points to account for long term drifts of the cell pressure during the experiments.

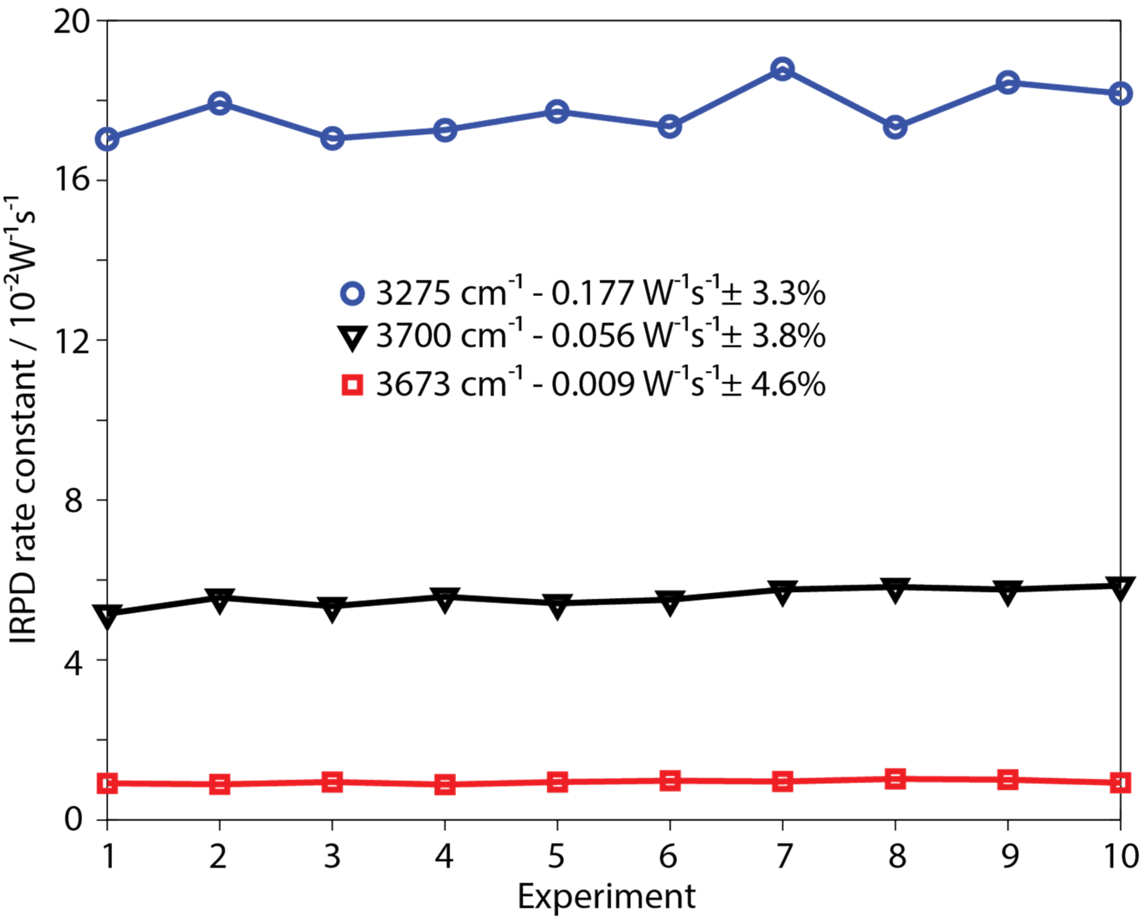

Figure S6. Experimental reproducibility for $[Gdm(H_2O)_{50}]^+$ at 133 K and three predefined wavelength in the free OH (3700 cm^{-1}, black) and bonded OH (3275 cm^{-1}, blue) region as well as for an intermediate wavenumber (3673 cm^{-1}, red) where only little dissociation is observed. Ten consecutive experiments were performed for each wavelength. The mean IRPD rate constant and the relative standard deviation are 0.056 $W^{-1}s^{-1}$±3.8% (3700 cm^{-1}, blue), 0.177 $W^{-1}s^{-1}$±3.3% (3275 cm^{-1}, blue) and 0.009 $W^{-1}s^{-1}$ ±4.6% (3673 cm^{-1}, blue), respectively.

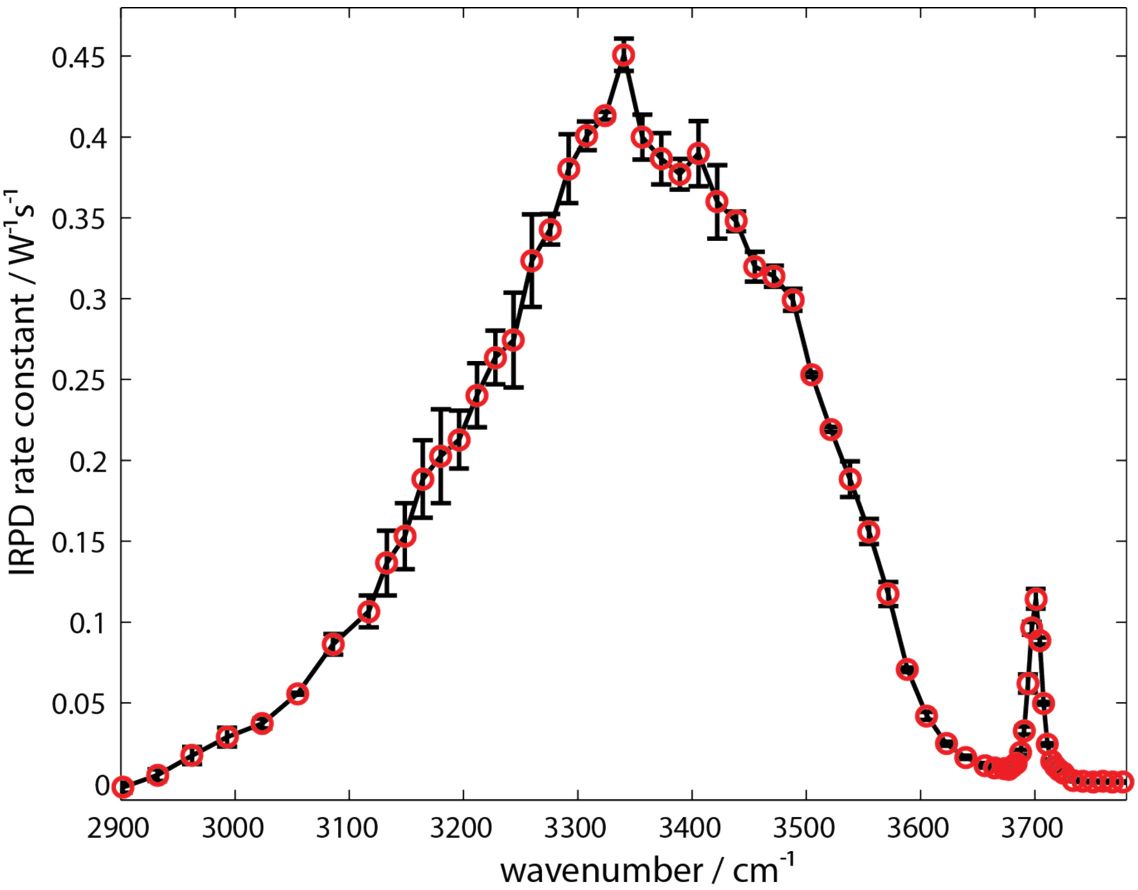

Figure S7. Experimental reproducibility of the full IRPD spectrum of $[Gdm(H_2O)_{100}]^+$ at 133 K. On four days the full IRPD spectrum of $[Gdm(H_2O)_{100}]^+$ was measured, tuning the OPO/OPA every time to identical wavelength starting at ~ 3780 cm^{-1} . The data

points represent the mean of the measurements and the error bars indicate the standard deviation for every data point. The calculated relative uncertainty of I(fOH)/I(HB) for this spectrum is ±8%.

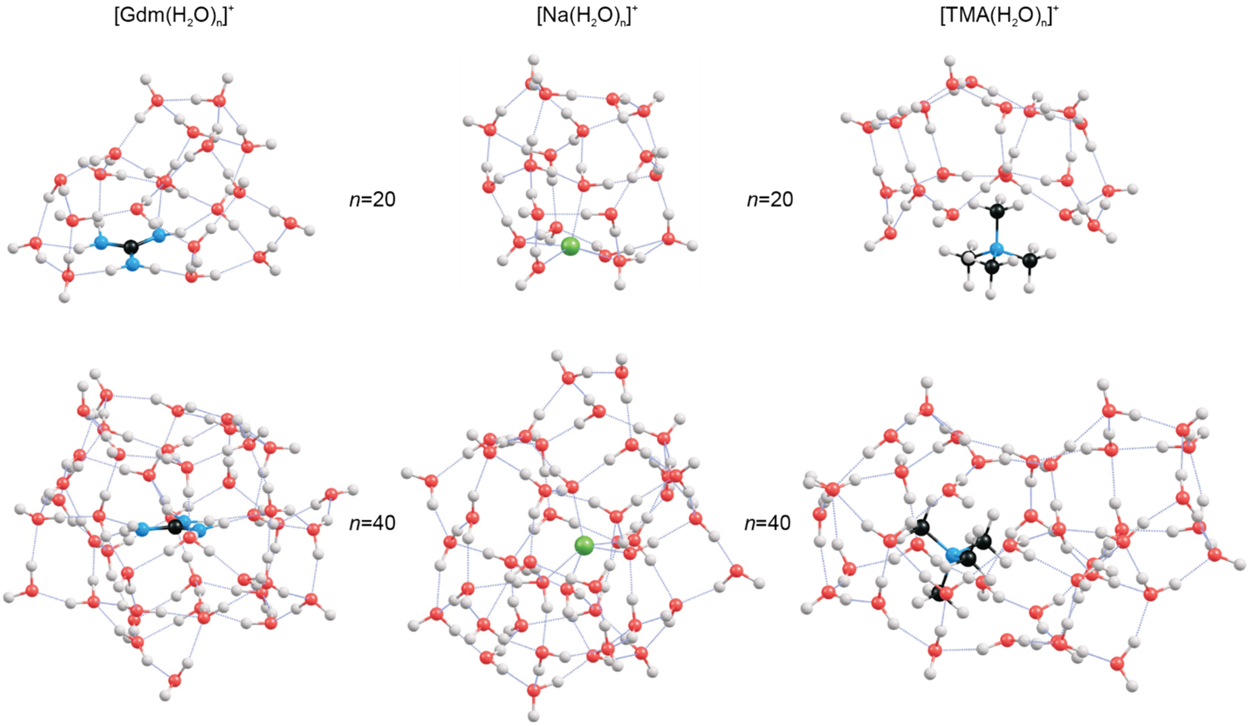

Figure S8. Representative structures of $[Gdm(H_2O)_n]^+$, $[Na(H_2O)_n]^+$ and $[TMA(H_2O)_n]^+$ obtained from B3LYP/6-31++G** calculations. Oxygen, hydrogen, carbon, nitrogen and sodium atoms are shown as red, white, black, blue and green spheres, respectively.

Table S1. RMSD value of the HB region of IRPD spectra between 2900-3630 cm^{-1} for Na^+ and TMA^+ clusters with respect to Gdm^+ clusters of the same size.

Size	Na^+	TMA^+	$(Na^+/TMA^+)-1$ / %
20	0.1537	0.1028	50
30	0.1336	0.0593	125

40	0.1060	0.0587	81
50	0.0796	0.0708	12
75	0.0790	0.0783	1
100	0.0497	0.0470	6
